# Mitotic Activation of a Novel Histone Deacetylase 3-Linker Histone H1.3 Protein Complex by Protein Kinase CK2*

**DOI:** 10.1074/jbc.M115.643874

**Published:** 2015-12-09

**Authors:** Hemangi Patil, Carrie Wilks, Rhiannon W. Gonzalez, Sudheer Dhanireddy, Heather Conrad-Webb, Michael Bergel

**Affiliations:** From the Department of Biology, Texas Woman's University, Denton, Texas 76204

**Keywords:** chromatin, histone deacetylase 3 (HDAC3), mitosis, mitotic spindle, protein kinase, linker histone H1.3, protein kinase CK2

## Abstract

Histone deacetylase 3 (HDAC3) and linker histone H1 are involved in both chromatin compaction and the regulation of mitotic progression. However, the mechanisms by which HDAC3 and H1 regulate mitosis and the factors controlling HDAC3 and H1 activity during mitosis are unclear. Furthermore, as of now, no association between class I, II, or IV (non-sirtuin) HDACs and linker histones has been reported. Here we describe a novel HDAC3-H1.3 complex containing silencing mediator of retinoic acid and thyroid hormone receptor (SMRT) and nuclear receptor corepressor 1 (N-CoR) that accumulated in synchronized HeLa cells in late G_2_ phase and mitosis. Nonetheless, the deacetylation activity by HDAC3 in the complex was evident only in mitotic complexes. HDAC3 associated with H1.3 was highly phosphorylated on Ser-424 only during mitosis. Isolation of inactive HDAC3-H1.3 complexes from late G_2_ phase cells, and phosphorylation of HDAC3 in the complexes at serine 424 by protein kinase CK2 (also known as casein kinase 2) activated the HDAC3 *in vitro. In vivo*, CK2α and CK2α' double knockdown cells demonstrated a significant decrease in HDAC3 Ser-424 phosphorylation during mitosis. HDAC3 and H1.3 co-localized in between the chromosomes, with polar microtubules and spindle poles during metaphase through telophase, and partially co-localized with chromatin during prophase and interphase. H1 has been reported previously to associate with microtubules and, therefore, could potentially function in targeting HDAC3 to the microtubules. We suggest that phosphorylation of HDAC3 in the complex by CK2 during mitosis activates the complex for a dual role: compaction of the mitotic chromatin and regulation of polar microtubules dynamic instability.

## Introduction

Histone deacetylases (HDACs)^2^ are a class of enzymes responsible for the deacetylation of core histone tails and non-histone proteins. The removal of acetyl groups from core histone tails leads to an increase in their positive charge and, therefore, a stronger ionic interaction between the core histone tails and the negatively charged DNA. This, in turn, increases the compaction of chromatin and the formation of the 30-nm fiber, resulting in transcriptional repression (1). Although HDACs play an important role in the maintenance of the epigenetic landscape through their activity on core histones, HDACs also target non-histone proteins, both functions that contribute to cell proliferation and differentiation (2, 3).

Histone deacetylase 3 (HDAC3) has been shown recently to play key roles in mitotic progression. HDAC3 activity is required for the global deacetylation necessary for chromatin compaction into mitotic chromosomes (4). In addition, loss of functional HDAC3 resulted in mitotic spindle collapse, chromosomal misalignment (5), impaired microtubule-to-kinetochore attachment (5), and premature spindle assembly checkpoint activation (6). Fadri-Moskwik *et al.* (6) have demonstrated that these mitotic defects could be explained by the failure of HDAC3 to activate Aurora B kinase by deacetylation in early mitosis.

Although linker histone H1 is an architectural protein, like HDACs, H1 plays a role in chromatin compaction, transcription repression, and mitotic regulation. The binding of H1 to the nucleosome results in a reduction of the entry-exit angle of DNA, leading to the stabilization of the 30-nm fiber. This compaction can limit the access of transcription factors to the DNA, resulting in transcriptional repression (7). The phosphorylation of linker histone H1 is also essential for the formation of mitotic chromosomes and mitotic progression. Treatment of cells with a kinase inhibitor led to elongated chromosomes that did not align properly in mitosis and were unable to separate at the onset of anaphase (8). Therefore, like HDAC3, the absence of phosphorylated H1 leads to abnormal alignment of mitotic chromosomes. Their similar phenotypes led to the question of whether there is a physical or functional association between HDACs and H1s in either transcriptional regulation or cell cycle control. In addition to phosphorylation, linker histone H1 can be acetylated (9, 10), although the function of this modification is not well understood. Vaquero *et al.* (9) have reported an interaction between H1 and the NAD^+^-dependent HDAC SirT1 that represses transcription through histone H4 Lys-16 deacetylation, H1 recruitment to the promoter, and demethylation of histone H3 Lys-79 (9). The interaction between linker histone H1 and SirT1 further suggests the potential for an interaction between other histone deacetylases and histone H1.

Here we show a novel stable association between HDAC3 and the linker histone subtype H1.3 in HeLa Cells. This complex includes the corepressors SMRT and N-CoR and at least four additional proteins. The abundance of this complex increased significantly in late G_2_ phase and into mitosis. The HDAC3 within the complex exhibited histone H3K9 deacetylase activity, which was dependent on mitosis and induced by HDAC3 phosphorylation at serine 424. *In vitro*, protein kinase CK2 was found to phosphorylate complexed HDAC3 at serine 424, leading to its activation in the complex. *In vivo*, CK2 knockdown cells demonstrated a significant reduction in HDAC3 phosphorylation at serine 424 during mitosis. HDAC3 and H1.3 co-localized mainly at the area of polar microtubules and spindle poles in mitotic HeLa cells, suggesting a potential role in the regulation of polar microtubule dynamics in mitosis.

## Experimental Procedures

### 

#### 

##### Immunoprecipitations and Western Blotting Analysis

HeLa S3 and MCF-7 cells were maintained in Dulbecco's modified Eagle's medium (Gibco/BRL) supplemented with 10% fetal bovine serum (Gemini) and 1% penicillin and streptomycin (Gibco/BRL). Cells were grown in the presence of 5% atmospheric CO_2_ and 100% humidity at 37 °C. Exponentially growing cells were harvested and washed with cold 1× PBS and lysed in co-immunoprecipitation assay buffer (0.5% Nonidet P-40, 0.8% 0.5 m NaF, 2% 100 mm sodium orthovanadate, and a mini complete protease inhibitor mixture tablet (Roche Life Science) in 1× PBS). After 30 min of incubation at 4 °C, cell lysates were passed through 20-gauge syringes and centrifuged for 20 min at 10,000 × *g* at 4 °C to collect the supernatant, which was used for immunoprecipitation assays and Western blotting analysis. The cell lysate was precleared with non-immune IgG or IgM and protein A/G-agarose or L-agarose beads (Santa Cruz Biotechnology), respectively. Immunoprecipitation was performed with anti-HDAC1–11 (Santa Cruz Biotechnology), anti-Histone H1 (Santa Cruz Biotechnology), and anti-phospho-H1 (Millipore) antibodies in a concentration of 1–2 μg/ml. Non-immune IgG/IgM (Santa Cruz Biotechnology) at the same final concentration was used as a negative control. After overnight incubation at 4 °C, immunocomplexes and protein beads were collected by centrifugation at 1000 × *g* at 4 °C for 5 min. The immunocomplexes were washed three times with radioimmune precipitation assay buffer, resuspended in 30 μl of SDS-PAGE loading buffer, and denatured by heating at 95 °C for 5 min. For immunoblotting, the proteins were resolved by SDS-PAGE (8% for HDAC3 and 12% for H1) and transferred onto PVDF membranes (Millipore). Membranes were probed overnight at 4 °C with one of the following primary antibodies: anti-HDAC3 (Santa Cruz Biotechnology), anti-Histone H1 (Santa Cruz Biotechnology), anti-actin (Sigma), anti-phospho-H3S10 (Upstate), anti-phospho-H1 (Abcam), anti-histone H1.1-H1.5 (Abcam), anti-SMRT (Santa Cruz Biotechnology), anti-N-CoR (Abcam), anti-acetyl-H3K9 (Millipore), anti-acetyl-H4K5 (Santa Cruz Biotechnology), anti-trimethyl-H3K9 (Millipore), anti-phosphoserine (Invitrogen-Zymed Laboratories Inc.), anti-HDAC3-P-S424 (Abcam), anti-CK2α subunit (Abcam), and anti-CK2α' subunit (Abcam). The membrane was then incubated with horseradish peroxidase-conjugated secondary antibody, and proteins were visualized using an ECL Plus kit (Amersham Biosciences) or incubated with LI-COR IRDye 800CW secondary antibody and scanned with a LI-COR Odyssey CLX imager.

##### Pulldown Assays

Human recombinant HDAC3 (8 μg, Biomol) was incubated with human recombinant H1.3 (4 μg, Alexis Biochemicals). Pulldown was carried out using anti-HDAC3 antibody with protein A/G-agarose beads. After overnight incubation at 4 °C, the reactions were subjected to centrifugation at 1000 × *g* for 5 min at 4 °C. The complex was dissociated with addition of SDS-PAGE loading buffer and resolved on SDS-PAGE. The gel was stained with Coomassie Blue RX-250 (Bio-Rad), and the stained protein bands were analyzed by densitometry using Alpha Innotech and Fluorchem HD2 software.

##### Cell Synchronization and Flow Cytometric Analysis

Exponentially growing HeLa S3 cells were treated twice with 2 mm thymidine (Sigma) for 18 h, with 11-h release between the treatments to block cells in S phase. Early G_2_ phase cells were collected 3 h after release from S phase block, whereas late G_2_ phase cells were collected after 6 h of release. S phase cells were further treated with 100 nm nocodazole (Sigma) to arrest cells in mitosis. An aliquot of the synchronized cell population was fixed with 4% paraformaldehyde (Fisher) and analyzed with flow cytometry (BD FACSCalibur). The fixed cells were washed twice with 1× PBS, permeabilized with 0.25% Triton X-100 (Fisher), and blocked with 1% BSA (Sigma). Cells were stained with either mitosis-specific marker antibody, phospho-H3S10 conjugated with FITC (Millipore), or control antibody IgG-FITC (Millipore). Counterstaining with propidium iodide (BD Biosciences) was used for DNA analysis of the cell cycle. From each synchronized cells population, an aliquot of cells was washed twice with 1× PBS, and the whole cell extracts were prepared in radioimmune precipitation assay buffer for complex analysis using immunoprecipitation assays as described earlier.

##### Hyperacetylated Core Histone Isolation

HeLa S3 cells were treated with 2 μm of the HDAC inhibitor trichostatin A for 20 h. Cells were washed twice with ice-cold 1× PBS and lysed sequentially with lysis buffer A (10 mm Tris-HCl (pH 7.4), 10 mm NaCl, 3 mm MgCl_2_, 0.5% Nonidet P-40, and 1 mm PMSF) and then lysis buffer B (10 mm Tris-HCl (pH 7.4), 3 mm CaCl_2_, 2 mm MgCl_2_, 1% Nonidet P-40, and 1 mm PMSF). To isolate the nuclei, cells were disrupted with a Dounce homogenizer and pestle B. The nuclei were centrifuged at 500 × *g* for 8 min at 4 °C and resuspended in 0.25 m H_2_SO_4_. The sulfuric acid extract was passed through a Dounce homogenizer with pestle A and incubated on ice for 15 min. The acid extract was centrifuged at 4000 × *g* for 10 min at 4 °C. The supernatant was added to 6 volumes of ice-cold 100% acetone, incubated at −20 °C for 3 h, and centrifuged at 4000 × *g* for 10 min at 4 °C. The core histone pellet was washed twice with 95% acetone, dried, and reconstituted in deionized distilled water.

##### Hyperacetylated Mononucleosomes Isolation

HeLa S3 cells were treated with 2 μm trichostatin A for 20 h. The cell nuclei were isolated as described and subjected to micrococcal nuclease digestion. Histone H1 was removed from nucleosomes by incubation with CM Sephadex beads (40–100 μm, Sigma). Nucleosomes were dialyzed in a Slide-A-Lyzer cassette (10,000 molecular weight cutoff, Pierce) against sucrose dilution buffer (25 mm NaCl, 10 mm Tris-HCl (pH 7.5), and 0.1 mm EDTA) and loaded on continuous sucrose gradients (12–50%). The gradient was centrifuged at 100,000 × *g* for 24 h at 4 °C. Fractions (1 ml) were collected from the gradient and analyzed on agarose gel to detect and isolate mononucleosomes.

##### Histone Deacetylase Assay

Synchronized HeLa S3 cell extracts from late G_2_ phase and mitosis were immunoprecipitated as described earlier using one of the following antibodies: anti-HDAC3 (Santa Cruz Biotechnology), anti-histone H1 (Santa Cruz Biotechnology), anti-phospho-histone H1 (Upstate), or non-immune IgG (Santa Cruz Biotechnology). They were then mixed with protein A/G-agarose beads (Santa Cruz Biotechnology). Bead immunocomplexes were washed twice with radioimmune precipitation assay buffer, followed by a wash with HDAC buffer (10 mm Tris-HCl (pH 8.0), 10 mm NaCl, 10% glycerol, and complete mini protease inhibitor mixture (Roche Life Science)). The washed bead immunocomplexes were incubated with hyperacetylated mononucleosomes in HDAC buffer at 37 °C for 40 min. After the incubation, SDS-PAGE loading buffer was added to the reaction mixture, and the reaction mixture was resolved by 15% SDS-PAGE. Western blotting analysis was performed using anti-acetyl-H3K9 (Upstate) and anti-acetyl-H4K5 antibodies (Santa Cruz Biotechnology). The HDAC assay reaction with the recombinant HDAC3-SMRT complex (Cell Sciences) served as a positive control, whereas the reaction with non-immune IgG (Santa Cruz Biotechnology) served as a negative control.

##### Phosphorylation of the HDAC3-H1.3 Complex

The HDAC3-H1.3 immunocomplex from late G_2_ phase cell extract was subjected to phosphorylation with protein kinase CK2 (New England Biolabs) and 200 mm ATP with [γ-^32^P]ATP (500 μCi/mmol (PerkinElmer Life Sciences)). Reactions were performed at 30 °C for 30 min. An aliquot was resolved by SDS-PAGE. The gels were dried, exposed to a Kodak storage phosphor screen, and scanned with Bio-Rad Molecular Imager FX to confirm phosphorylation of HDAC3. The phosphorylated immunocomplex was used for further HDAC assay analyses.

##### siRNA Knockdown of CK2α and α'

3 × 10^5^ MCF-7 cells were seeded to each well in a 6-well culture dish with 5 nm siRNA against both CK2α and CK2α' (Ambion, catalog nos. s3638 and s3641, respectively) or with AllStar negative control scrambled siRNA (Qiagen) and 1.5% HiPerFect transfection reagent (Qiagen) according to the recommendations of Qiagen. The cells were incubated with transfection complexes for 48 h. Cells were lysed by addition of 100 μl of 1× SDS-PAGE loading buffer 96 h after plating. Mitotic arrest of siRNA knockdown cells was achieved by incubation of cells with 100 nm nocodazole for 19 h prior to lysis. The mitotic index was determined by plating 6.2 × 10^4^ cells/well in a 24-well culture dish with a glass coverslip along with 5 nm siRNA and 1.5% HiPerFect transfection reagent for 48 h. Nocodazole (100 nm) was added 19 h prior to staining. Cells were stained with DAPI and mounted with ProLong Diamond antifade (Invitrogen) 96 h after plating. The cells were imaged at ×40 magnification using a Zeiss Axiovert 200 M optical microscope. All cells in a field were counted, following a zig-zag pattern until at least 1500 cells were counted.

##### Immunofluorescence Microscopic Analysis

HeLa cells were grown in a 6-chamber slide (LabTek) to 80% confluence, washed with 1× PBS, and fixed with 4% paraformaldehyde (Fisher) for 10 min. Cells were treated with TNBS buffer (0.1% Triton X-100, 1% FBS, and 0.1% NaN_3_ in 1× PBS) for 20 min for permeabilization and blocking and then incubated overnight at 4 °C with one of the following primary antibodies: mouse anti-HDAC3 (Santa Cruz Biotechnology), rabbit anti-histone H1.3 (Abcam), or goat anti-Eg5 (Santa Cruz Biotechnology). Negative controls for all experiments were performed using non-immune IgG from the same species as the primary antibody. Following washes with 1× PBS, cells were incubated with secondary antibody: goat/donkey anti-mouse-FITC (Santa Cruz Biotechnology) or goat or donkey anti-rabbit-Texas Red (Santa Cruz Biotechnology) or donkey anti-goat-FITC or donkey anti-goat-TR for 1 h 15 min at room temperature. After the 1× PBS washes, chromosomes were stained with Hoechst (Invitrogen) for 5 min, and Prolong Antifade mounting medium (Invitrogen) was added before sealing the coverslip. The cells were imaged at ×1000 magnification using a Zeiss Axiovert 200 M optical microscope with confocal attachment, and the digital images were analyzed with ImageJ software.

##### Isolation and Identification of the Endogenous HDAC3-H1.3 Complex

Immunocomplexes from late G_2_ phase and mitosis cell extracts were obtained as described using anti-histone H1, anti-HDAC3, and non-immune IgG (Santa Cruz Biotechnology). These immunocomplexes were resolved on SDS-PAGE and stained with a silver stain kit (Pierce). Bands of interest were excised and destained for Western blotting analysis.

## Results

### 

#### 

##### Histone Deacetylase 3 Associates with Histone H1.3 in Vivo and in Vitro

**FIGURE 1.**
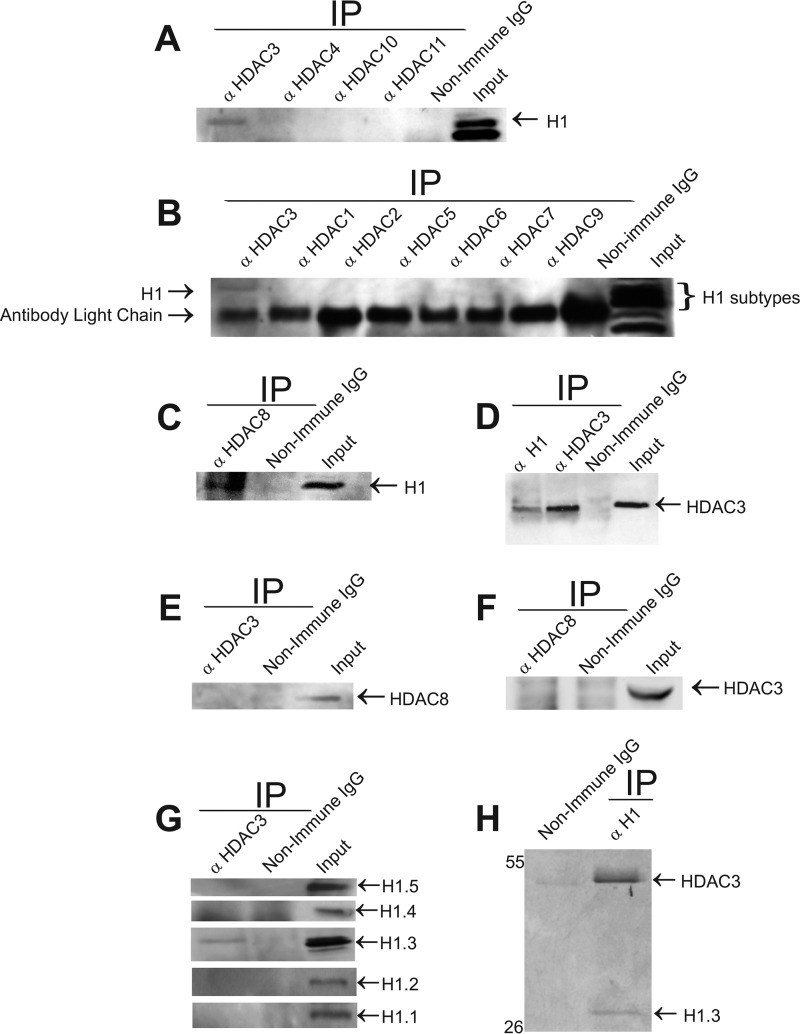
**Histone deacetylase 3 stably and directly associates with linker histone subtype H1.3.** *A–C*, HDAC3 interacts with histone H1 *in vivo*. HeLa S3 cell extracts were immunoprecipitated (*IP*) with anti-HDACs 1–11 and non-immune IgG as a negative control and monitored by Western blotting analysis for the presence of histone H1. Inputs represent 1.5% of the total protein used for immunoprecipitation in all experiments unless specified otherwise. *D*, anti-H1 antibody co-immunoprecipitates HDAC3. HeLa S3 whole cell extracts were used for immunoprecipitation with anti-histone H1, anti-HDAC3 (positive control), and non-immune IgG (negative control) and monitored by Western blotting analysis for the presence of HDAC3. *C* and *D*, HDAC8 interacts with histone H1 *in vivo. E* and *F*, HDAC3 and HDAC8 are not part of the same complex. Immunoprecipitates of anti-HDAC3 (*E*) and anti-HDAC8 (*F*) were analyzed to detect the presence of HDAC8 (*E*) or HDAC3 (*F*). *G*, HDAC3 interacts with histone variant H1.3. Co-immunoprecipitates with anti-HDAC3 were monitored by Western blotting analysis for presence of H1.1, H1.2, H1.3, H1.4, and H1.5. Only H1.3 appeared to associate with HDAC3. *H*, HDAC3 interacts directly with histone H1.3. Human recombinant HDAC3 and human recombinant histone H1.3 were mixed, subjected to pulldown with anti-HDAC3, and resolved on SDS-PAGE, followed by Coomassie Blue staining. Immunoprecipitation with non-immune IgG served as a negative control.

HDACs and linker histone H1 are known to play a role in the progression of mitosis and in chromatin compaction (4–6, 8). We therefore hypothesized that HDACs and H1 might cooperate in mitosis through their physical association. To explore the possible interaction between HDACs and Histone H1, we carried out a series of co-immunoprecipitation experiments with HeLa S3 extracts using antibodies against class I, II, and IV HDAC proteins. In all co-immunoprecipitations, non-immune IgG from the relevant species was used as a negative control. Western blotting analysis of the immunoprecipitates using an antibody against histone H1 revealed a stable association of histone H1 with HDAC3 and HDAC8 (Fig. 1, *A–C*) but not with the remaining HDACs (1, 2, 4, 5, 6, 7, 9, 10, and 11) (Fig. 1, *A* and *B*). When anti-H1 antibody was used for the reciprocal immunoprecipitations and anti-HDACs antibodies for the Western blotting analysis, an association could only be confirmed between H1 and HDAC3 (Fig. 1*D*). To investigate the possibility of HDAC3, HDAC8, and histone H1 existing as part of the same complex, we performed co-immunoprecipitations of HDAC3, followed by Western blotting with anti-HDAC8 antibody and vice versa. The resulting Western blotting analysis did not demonstrate an HDAC3 and HDAC8 association in the same complex (Fig. 1, *E* and *F*). Consequently, we focused on the association of HDAC3 with histone H1 for further analysis. To identify whether a specific histone H1 subtype was associated with HDAC3, we carried out co-immunoprecipitation assays using anti-HDAC3 antibody, followed by Western blotting analysis using antibodies for linker histone subtypes H1.1, H1.2, H1.3, H1.4, and H1.5. The Western blotting analysis indicated a specific association only between HDAC3 and histone H1.3 (Fig. 1*G*).

To determine whether the interaction between HDAC3 and histone H1.3 is direct, we performed *in vitro* pulldown experiments using recombinant human HDAC3 and H1.3 proteins. The recombinant human HDAC3 was incubated with recombinant human H1.3. The pulldown was carried out using an anti-HDAC3 antibody, and the complex was resolved on SDS-PAGE and Coomassie-stained to visualize proteins. The results were consistent with the co-immunoprecipitation assays and demonstrated that HDAC3 binds directly to histone H1.3 (Fig. 1*H*).

##### Interaction of HDAC3 with Histone H1.3 Increases during Late G_2_ Phase and Mitosis

**FIGURE 2.**
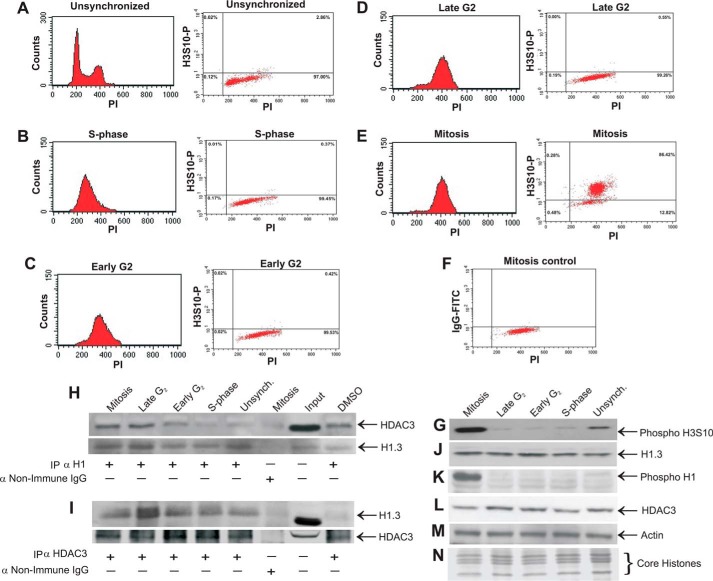
**Interaction of HDAC3 with histone H1.3 increases during late G_2_ phase and mitosis.** HeLa S3 cells synchronized by double thymidine block and nocodazole treatment were analyzed by flow cytometry to confirm the isolation of synchronized S phase, early G_2_ phase, late G_2_ phase, and mitotic cells. *A*, histogram (propidium iodide (*PI*) staining) and dot plot (phospho-H3S10-FITC and PI staining) of unsynchronized HeLa S3 cells. The histogram shows G_0_/G_1_ (first peak), G_2_/M (second peak), and S phase (cell population in the middle of the two peaks). The dot plot shows 2.86% mitosis in unsynchronized cells. *B–F*, histogram and dot plot of HeLa S3 cells synchronized to S phase using a double thymidine block (*B*), early G_2_ phase after 3-h release from the block (*C*), late G_2_ phase after 6-h release from the block (*D*), and mitosis after 3 h release from the block, followed by nocodazole treatment (*E*). *F*, a mitosis control dot plot served as a negative control for staining of the mitotic populations with IgG-FITC and PI. HeLa S3 cells synchronized to S phase, early G_2_ phase, late-G_2_ phase, and mitosis using a double thymidine block and nocodazole treatment were harvested in immunoprecipitation lysis buffer and immunoprecipitated with antibodies to anti-histone H1 (*H*) and anti-HDAC3 (*I*) and then subjected to Western blotting analysis with anti-HDAC3 and anti-histone H1.3 antibodies (*H* and *I*, respectively). A similar immunoprecipitation, followed by Western blotting against the precipitated protein, was used in each case to verify the close to equal immunoprecipitation of both H1.3 (*H*) and HDAC3 (*I*). Synchronized cell extracts and unsynchronized (*Unsynch*) control extracts were analyzed by Western blotting for protein levels using the following antibodies: anti-phospho-H3S10 (*G*), anti-histone H1.3 (*J*), anti-phospho-H1 (*K*), anti-HDAC3 (*L*), and anti-actin (*M*). Coomassie staining of core histones (*N*) and immunoblot with anti-actin (*M*) served as loading controls. *DMSO*, dimethyl sulfoxide.

Our working hypothesis was that this HDAC3-H1.3 complex may participate in the regulation of mitosis. If this assumption is correct, we would expect to find higher levels of the complex during or just before mitosis. To examine HDAC3-H1.3 complex formation during various cell cycle stages, HeLa S3 cells were synchronized in S phase, early G_2_ phase, late G_2_ phase, and mitosis using a double thymidine block followed by nocodazole treatment. The synchronization to these various stages was determined by flow cytometry with propidium iodide staining for DNA content and FITC anti-phospho-H3S10 as a mitotic marker (Fig. 2, *A–F*). Western blotting further confirmed the synchronization and demonstrated that the levels of the mitotic marker phospho-H3S10 were greatly enriched in mitotic cells compared with unsynchronized cells, and they were significantly higher in mitotic lysates compared with the late G_2_, early G_2_, and S phase cell lysates (Fig. 2*G*).

Co-immunoprecipitation assays were carried out on protein extracts from each of these cell cycle phases using antibodies against either histone H1 (Fig. 2*H*) or HDAC3 (Fig. 2*I*), followed by Western blotting analysis for detection of HDAC3 (Fig. 2*H*) or H1.3 (Fig. 2*I*). Equal precipitation of H1.3 and HDAC3 was demonstrated by running parallel immunoprecipitations followed by Western blotting with anti H1.3 antibody (Fig. 2*H*) or anti HDAC3 antibody (Fig. 2*I*). The results indicated a significantly higher association between HDAC3 and histone H1 during late G_2_ phase and mitosis. To elucidate whether the increase in the level of H1.3-HDAC3 complex during late G_2_ phase and mitosis is associated with an increase in the level of components of this complex, we examined the levels of histone H1.3 (Fig. 2*F*), phospho-histone H1 (Fig. 2*G*), and HDAC3 (Fig. 2*H*) proteins by Western blot analysis. The levels of HDAC3 and histone H1.3 were unaltered during the various cell cycle stages studied. Phosphorylation of histone H1 was higher during mitosis, as reported previously (11, 12). Therefore, the increase in the level of the complex cannot be attributed to the increase in the level of H1.3 or HDAC3 during late G_2_ phase or mitosis. The increase in the complex level can also not be attributed to the increase in phospho-H1 because the increase in the complex level occurred during late G_2_ phase, when the levels of phospho-H1 were still low.

##### Endogenous HDAC3, Associated with H1.3 in a Complex, Is Functionally Active as Deacetylase When Isolated from Mitotic but Not from Late G_2_ Phase Cells

**FIGURE 3.**
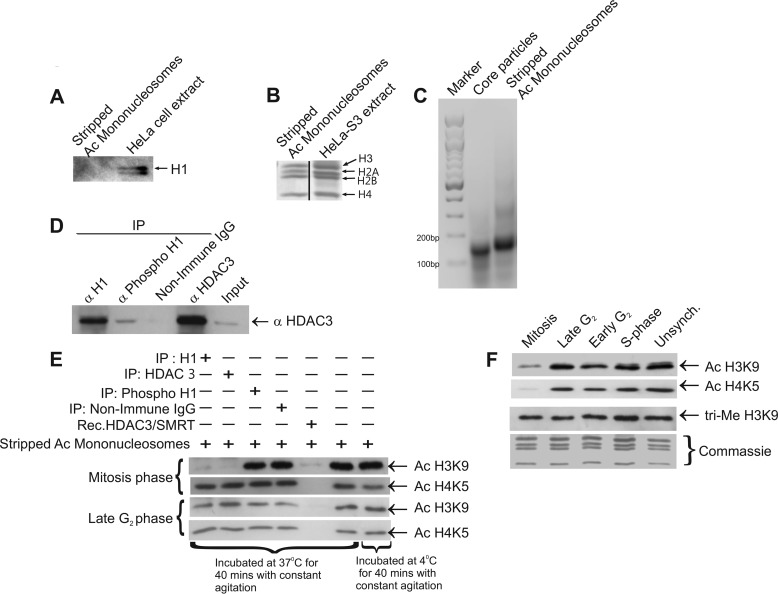
**Late G_2_ phase and mitotic deacetylation activity of HDAC3 complexed with histone H1.3.** *A*, Western blotting analysis to ensure the absence of histone H1 from acetylated (*Ac*) mononucleosomes used for the HDAC assay. *B*, Coomassie staining of isolated acetylated and mononucleosomes showing the presence of core histones in equimolar ratios. *C*, agarose gel (2%) demonstrating the intact DNA size (about 180 bp) of the isolated acetylated mononucleosomes. *D*, phosphorylated HDAC3-H1.3 complexes were reimmunoprecipitated (*IP*) using anti-histone H1, anti-phospho-H1, anti-HDAC3, and non-immune IgG and analyzed by Western blotting for verification of the presence of HDAC3 as a control before the HDAC assay. *E*, the deacetylation activity of the HDAC3-H1.3 complex was assayed upon immunoprecipitation with anti-histone H1, anti-phospho-H1, and a positive control of anti-HDAC3 from mitotic or late G_2_ phase HeLa S3 whole cell extracts. Deacetylation in the presence of the recombinant (*Rec*) HDAC3/N-CoR2 complex served as another positive control, whereas non-immune IgG immunoprecipitate served as a negative control. Deacetylation of H3K9 and H4K5 in hyperacetylated mononucleosomes (isolated as described under “Experimental Procedures”) was assessed by Western blotting analysis after the deacetylation assay. *F*, acetylation levels of H3K9 and H4K5 and trimethylation (*tri-Me*) levels on H3K9 during various cell cycle stages as analyzed by Western blotting. Coomassie staining of core histones served as a loading control. *Unsynch*, unsynchronized.

To characterize the deacetylase activity of complexed HDAC3 *in vitro*, we investigated its ability to deacetylate known HDAC3 substrates, acetylated histone H3K9, and acetylated histone H4K5 in isolated mononucleosomes (2, 13). Hyperacetylated mononucleosomes were isolated by sucrose gradient and stripped of histone H1 by incubation with Sephadex beads, followed by dialysis. The nucleosomes were then tested for the absence of histone H1 by immunoblotting with anti-histone H1 antibody (Fig. 3*A*). Mononucleosomes were also tested for the integrity of all core histones by Coomassie staining (Fig. 3*B*) and for the presence of a linker DNA region (full-size nucleosome DNA) by agarose gel electrophoresis (Fig. 3*C*). HDAC3-H1.3 complexes were isolated by co-immunoprecipitation with antibody against histone H1, HDAC3, or phospho-histone H1, and the presence of HDAC3 in the complex was confirmed by Western blotting with anti-HDAC3 antibody (Fig. 3*D*). Another aliquot of these precipitated complexes was then tested for deacetylation ability when incubated with hyperacetylated mononucleosomes. Incubation with HDAC3-H1.3 complex isolated from mitotic cells showed the deacetylation of H3K9 (Fig. 3*E*, *first blot*) but not of H4K5 (Fig. 3*E*, *second blot*). Interestingly, late G_2_ phase cell complexes failed to deacetylate either substrate (Fig. 3*E*, *third* and *fourth blots*). Hyperacetylated mononucleosomes alone were incubated at the reaction assay temperature (37 °C) to verify the absence of intrinsic HDAC activity in the nucleosome preparation (Fig. 3*E*). Recombinant HDAC3-SMRT complex deacetylated both H3K9 and H4K5, demonstrating that the H1.3-HDAC3 complex has a distinct substrate specificity from rHDAC3-SMRT (Fig. 3*E*). As opposed to the HDAC3-H1.3 complex precipitated by anti-H1, the complex precipitated by anti-phospho-H1 antibody did not show significant deacetylation activity (Fig. 3*E*). This is consistent with the very low levels of HDAC3 that could be co-precipitated by the anti-phospho-H1 antibody in contrast to the anti-H1 antibody (Fig. 3*D*). The reason for the lower recovery of HDAC3 using anti-phospho-H1 antibody may be that the H1.3 subtype is phosphorylated to a lower level and at fewer sites (including during mitosis) in comparison with other H1 subtypes (11, 12). Alternatively, the anti-phospho-H1 antibody may have a lower affinity for phospho-H1.3 relative to the other phosphorylated H1 subtypes, resulting in a relatively lower yield of precipitated phospho-H1.3 and, therefore, less co-immunoprecipitated HDAC3. Another possibility may be a lower affinity of HDAC3 for phospho-H1.3.

To examine whether H3K9 and H4K5 are actually hypoacetylated in HeLa S3 cells during mitosis, we next tested the synchronized cell extracts by Western blotting analysis using antibodies against acetylated H3K9 and acetylated H4K5 (Fig. 3*F*). The resulting immunoblots indicated a significant reduction in acetylation of these residues during mitosis, consistent with the hypoacetylation of H3K9 and H4K5 during mitosis in SK-N-SH cells and human fibroblast cells reported previously (14). We suggest that the novel complex of HDAC3 associated with histone H1.3 may be involved in deacetylation of H3K9 during mitosis. Because deacetylation and methylation of core histone residues can be regulated coordinately (15), we further investigated whether deacetylation of H3K9 during mitosis may contribute to its trimethylation, which is a well known marker for heterochromatinization (16–18). When synchronized cell extracts were analyzed, the immunoblots indicated no change in the trimethylation levels of H3K9 (Fig. 3*F*).

##### The HDAC3-H1.3 Complex Contains SMRT and N-CoR

**FIGURE 4.**
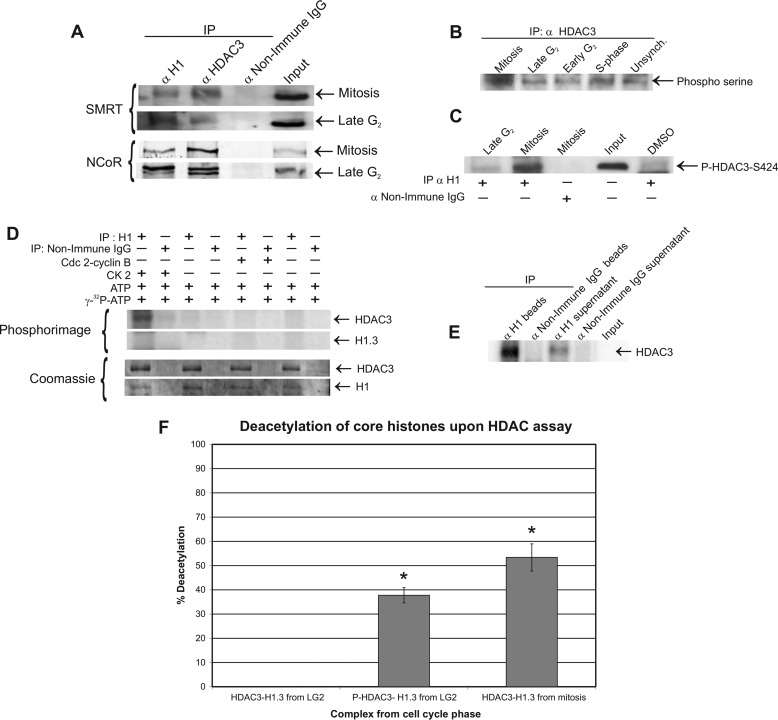
**HDAC3 phosphorylation by CK2 induces the deacetylation activity of the HDAC3-H1.3 complex *in vitro*.** *A*, endogenous SMRT and N-CoR associate with the HDAC3-H1.3 complex. HeLa S3 whole cell extracts of mitosis (*top blots*) and late G_2_ phase (*bottom blots*) were immunoprecipitated (*IP*) with anti-histone H1, anti-HDAC3, and non-immune IgG and monitored by Western blotting analysis for the presence of SMRT and N-CoR. *B*, serine residues on HDAC3 were hyperphosphorylated in mitotic HeLa S3 cell extracts, as detected by Western blotting analysis. *Unsynch*, unsynchronized. *C*, histone H1 immunoprecipitates from late G_2_ phase and mitosis were monitored for the presence of phospho-HDAC3-Ser-424 on immunoblot. Immunoprecipitate with non-immune IgG served as a negative control. *DMSO*, dimethyl sulfoxide. *D*, phosphorimage of *in vitro* phosphorylated HDAC3-H1.3 complex by Cdc2-cyclin B or protein kinase CK2 (using 500 μCi/mmol [γ-^32^P]ATP) on immunoprecipitates of anti-histone H1 and non-immune IgG. The levels of the immunoprecipitated H1 and the co-immunoprecipitated HDAC3 from the same gel was verified by Coomassie staining. *E*, HDAC3 remains associated with H1.3 and bound to the beads after *in vitro* phosphorylation by CK2 enzyme. The association of HDAC3 in the immunoprecipitated HDAC3-H1.3 complex after phosphorylation was assessed by 8% SDS-PAGE separation of proteins that remained bound to the beads and those proteins found in the supernatant after reaction with CK2. [^32^P]HDAC3 was detected by phosphorimage. *F*, deacetylation activity of the CK2-phosphorylated HDAC3-H1.3 complex isolated from late G_2_ phase cells, the late G_2_ phase complex without phosphorylation, and the mitotic complex. The deacetylation activity of the HDAC3-H1.3 complex was assayed upon immunoprecipitation with anti-histone H1 antibody as in Fig. 3*E*. The deacetylation of H3K9-Ac residue on isolated nucleosomal cores was examined by Western blotting analysis after the deacetylation assay. The graph represents the average percentage of deacetylation (*n* = 3). *Error bars* are mean ± S.E. *, *p* ≤ 0.05 (indicates a significant difference from the deacetylation activity of the late G_2_ phase complex; Friedman nonparametric analysis followed by pairwise comparison).

The next question asked was this: what is the mechanism that activates HDAC3 in the HDAC3-H1.3 complex at the transition of cells from G_2_ phase to mitosis? Activation of HDAC3 by its association with SMRT or N-CoR has been well documented (19, 20). To examine whether SMRT or N-CoR are associated with the isolated complex and whether their presence is altered at the late G_2_ phase-to-mitosis transition, we immunoprecipitated histone H1 and HDAC3 from late G_2_ phase and mitotic lysates, followed by Western blotting analysis for the presence of SMRT or N-CoR (Fig. 4*A*). The immunoblots showed that SMRT and N-CoR were associated with both HDAC3 and H1. However, both SMRT and N-CoR were present during mitosis and late G_2_ phase, and, therefore, could not explain the activation of the complex during mitosis. It is interesting to note that the late G_2_ phase complex, unlike the mitotic complex, contained a doublet that may be N-CoR splice variants, N-CoRω and N-CoRδ, which have been reported previously to have a functional significance in adipogenesis (21).

##### Phosphorylation of HDAC3 by Protein Kinase CK2 Up-regulates Its Deacetylation Activity In the HDAC3-H1.3 Complex

Our previous experiments have shown that the deacetylase activity of the HDAC3-H1.3 complex is up-regulated during mitosis but not in late G_2_ phase (Fig. 3, *A* and *B*). Zhang *et al.* (22) have shown that HDAC3 could be activated by phosphorylation of Ser-424 by CK2 *in vitro* and that Ser-424 on HDAC3 is a target for phosphorylation *in vivo*. However, CK2 phosphorylation of HDAC3 Ser-424 has not been shown *in vivo*, nor was it linked to mitosis. To explore the possibility that phosphorylation of the complexed HDAC3 by CK2 contributes to its activation during the transition from late G_2_ phase to mitosis, we first investigated whether HDAC3 is highly phosphorylated during mitosis. HDAC3 was immunoprecipitated from cell cycle-synchronized extracts, followed by Western blotting analysis with anti-phospho-serine antibody. Higher serine residue phosphorylation levels of HDAC3 were present in mitotic cells relative to late G_2_ and S phase cells (Fig. 4*B*).

To test whether Ser-424 in the *in vivo* complexed HDAC3 is being phosphorylated at a higher level during mitosis compared with late G_2_ phase, we immunoprecipitated the complex from late G_2_ phase and mitotic cell extracts using anti-histone H1 antibody followed by Western blotting analysis with anti-phospho-HDAC3-Ser-424 antibody. The resultant immunoblot clearly demonstrated higher phosphorylation of Ser-424 on HDAC3 associated with H1.3 during mitosis compared with late G_2_ phase (Fig. 4*C*). We next investigated whether CK2 can phosphorylate HDAC3 in the HDAC3-H1.3 complex obtained from late G_2_ phase cell extracts *in vitro* and whether this phosphorylation activates HDAC3. Immunoprecipitates obtained using anti-histone H1 antibody from late G_2_ phase extracts were incubated with CK2 in the presence of [γ-^32^P]ATP. The resulting phosphorimage demonstrated that CK2 could specifically phosphorylate HDAC3 from the HDAC3-H1.3 complex obtained from late G_2_ phase cell extracts (Fig. 4*D*, *top blots*). We also sought to determine whether CK2 can phosphorylate histone H1.3 from the complex. However, no band was detected on the phosphorimage in the H1 molecular weight range (Fig. 4*D*, *bottom blots*).

To evaluate the possibility that mitotic kinase Cdc2-cyclin B was also involved in phosphorylation of HDAC3, the assay was performed using recombinant Cdc2-cyclin B as well. The results demonstrated that Cdc2-cyclin B was unable to phosphorylate the HDAC3-H1.3 complex (Fig. 4*D*). Earlier reports have indicated the possibility that HDAC3 dissociates from its complexes when highly phosphorylated (22). To analyze whether HDAC3 dissociated from the HDAC3-H1.3 complex after *in vitro* phosphorylation by CK2, immobilized HDAC3-H1.3 complex was phosphorylated by CK2 while attached to agarose beads. After incubation, the proteins attached to the beads, and the proteins from the supernatant were resolved on SDS-PAGE. The resultant phosphorimage clearly indicated that a significant amount of the HDAC3 remained bound to the complex after phosphorylation (Fig. 4*E*), suggesting that CK2 phosphorylation does not result in dissociation of HDAC3 from the complex.

To evaluate whether the phosphorylation of HDAC3 by CK2 contributed to the activation of this complex during mitosis, histone H1 immunoprecipitates from late G_2_ phase extracts with and without *in vitro* phosphorylation by CK2 were incubated with acetylated mononucleosomes. The deacetylation ability of the immunoprecipitated complex was evaluated by examining the level of deacetylation of nucleosomal H3K9-Ac. Immunoprecipitated complexes from mitotic cells were used as a positive control for the deacetylation of nucleosomal H3K9-Ac. In three independent experiments, HDAC3-H1.3 complex from late G_2_ phase failed to deacetylate H3K9-Ac (0% acetylation). However, after *in vitro* phosphorylation by CK2, HDAC3-H1.3 complex deacetylated 38% of H3K9-Ac (*p* ≤ 0.006), a level that was comparable with the 53% deacetylation demonstrated by the mitotic H1.3-HDAC3 complex (Fig. 4*F*). These results suggested that HDAC3 activity in the HDAC3-H1.3 complex is activated by CK2 phosphorylation of HDAC3 in the complex upon entry of the cells into mitosis.

**FIGURE 5.**
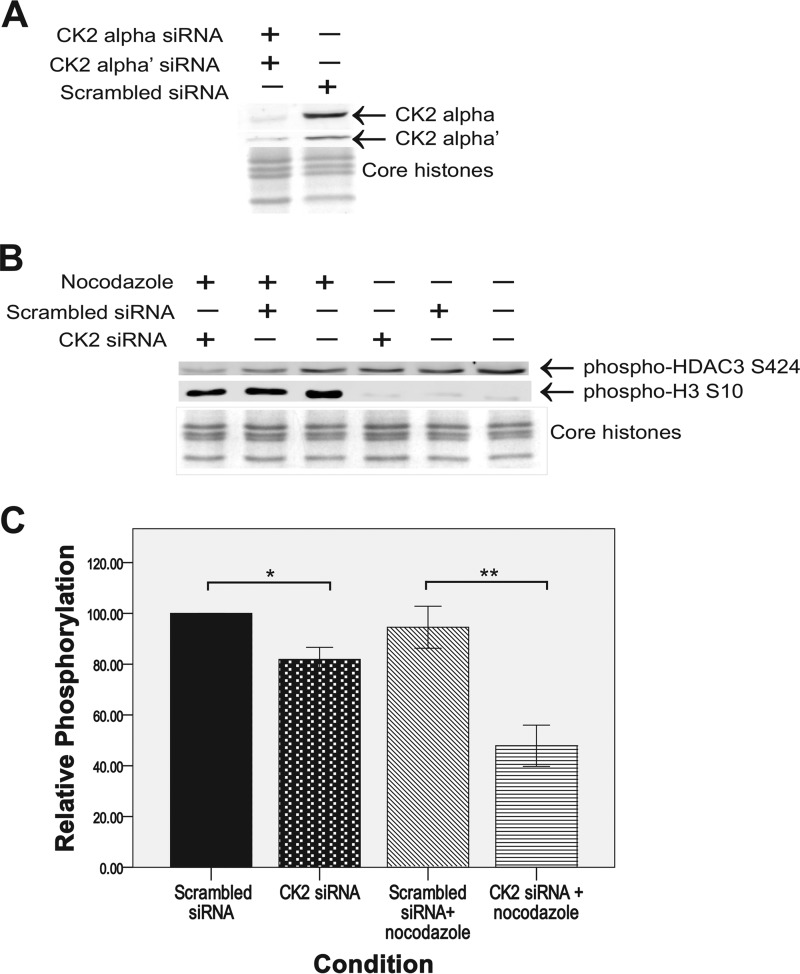
**Knockdown of CK2α and α' subunits decreases HDAC3 Ser-424 phosphorylation levels *in vivo*.** *A*, verification of ≥80% knockdown of both CK2α and α subunits in MCF-7 cells after siRNA treatment compared with scrambled siRNA treatment. The whole cell protein extracts were resolved on a 12% SDS-PAGE, and Western blotting with anti-CK2α and anti-CK2α' antibodies was performed. The loading control of core histones was resolved on 15% SDS-PAGE and stained with Coomassie Blue. *B*, Western blotting analysis of phosphorylation levels of HDAC3 Ser-424 after siRNA knockdown of CK2α and α subunits with and without mitotic arrest by nocodazole. Western blotting against phospho-H3 Ser-10 was used to verify mitotic arrest by nocodazole. All proteins in were resolved on 15% SDS-PAGE, including the core histones loading control gel. *C*, average percent phosphorylation of HDAC3 Ser-424 relative to cells treated with scrambled siRNA that were not arrested in mitosis (*n* = 3). *Error bars* are mean ± S.E. *, *p* ≤ 0.002; **, *p* ≤ 0.004 (Kruskal-Wallis nonparametric analysis followed by pairwise comparison).

To test whether CK2 significantly phosphorylates HDAC3 at serine 424 during mitosis, MCF-7 cells doubly knocked down for CK2α and CK2α', the catalytic subunits of the CK2 tetramer, were generated with siRNA. (MCF-7 cells demonstrated significant levels of the HDAC3-H1.3 complex (data not shown).) CK2α and CK2α' levels both decreased by 80% in the knockdown cells compared with the negative control (scrambled siRNA) (Fig. 5*A*). The knockdown cells were obtained both with and without mitotic arrest by nocodazole and analyzed by Western blotting with anti-phosopho-HDAC3-Ser-424 antibody. The immunoblots showed a statistically significant decrease of 49% in HDAC3 phosphorylation at Ser-424 in the mitotically arrested CK2α/CK2α' knockdowns compared with cells treated with nonspecific scrambled siRNA (Fig. 5, *B* and *C*). We also detected a significant 18% decrease of Ser-424 phosphorylation in unsynchronized CK2α/α' knockdown cells relative to unsynchronized control cells treated with nonspecific scrambled siRNA (Fig. 5, *B* and *C*). The mitotic index of the mitotically arrested cells (CK2α/α' knockdown) was 39.8% compared with the mitotic index of 2.3% of the unsynchronized cells (CK2α/α' knockdown). Considering both the mitotic index of 39.8% and the 49% inhibition of HDAC3 phosphorylation in mitotic CK2 α/α' knockdown cells, this indicated that most, if not all, of HDAC3-Ser-424 phosphorylation in mitotic cells was catalyzed by CK2.

##### HDAC3 and H1.3 Are Colocalized with Polar Microtubules and Spindle Poles during Metaphase, Anaphase, and Telophase

**FIGURE 6.**
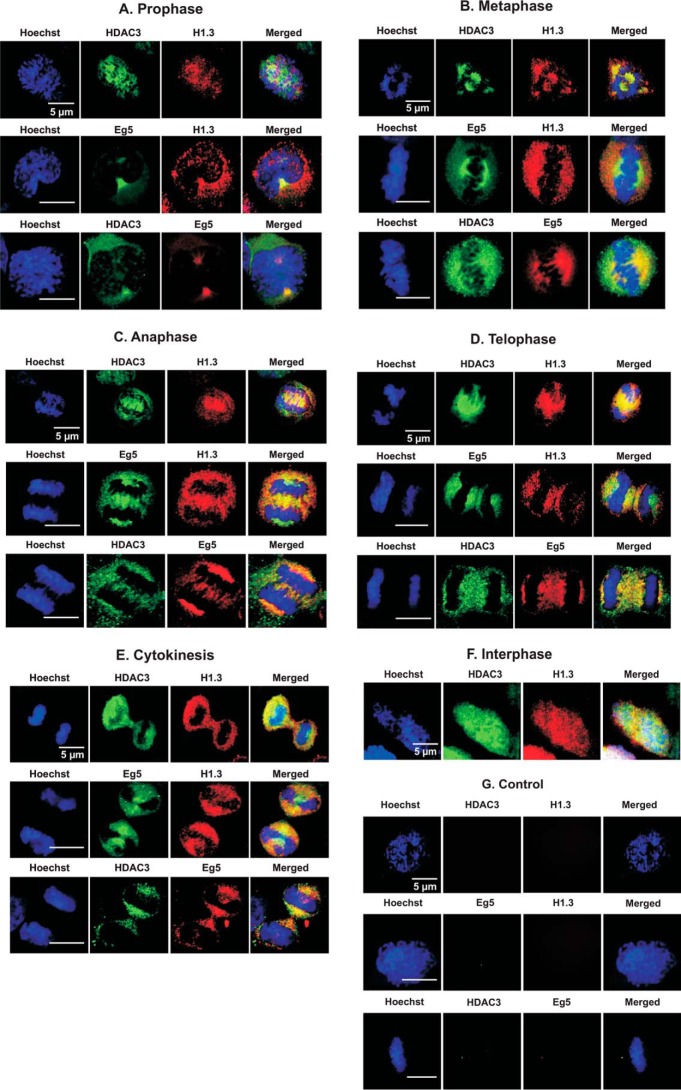
**HDAC3 and H1.3 co-localize with polar microtubules and centrosomes during metaphase-telophase stages and partially co-localizes during interphase and prophase.** HeLa cells from the various mitotic stages and interphase were subjected to indirect immunofluorescence staining. The antibodies used were anti-HDAC3 (*green*), anti-histone H1.3 (*red*), and Eg5 (*green/red*) as well as Hoechst (*blue*), using confocal imaging. Negative controls for all the immunostaining were performed using non-immune IgG as a primary antibody.

To localize HDAC3 and H1.3 during mitosis, HeLa cells were analyzed by immunofluorescent staining with anti-HDAC3 and anti-histone H1.3 antibodies using confocal microscopy. Consistent with the co-immunoprecipitation results, we found specific co-localization of HDAC3 and histone H1.3 proteins in most mitotic stages as well as partial co-localization in the nucleus of prophase and interphase cells (Fig. 6). During interphase, both HDAC3 and H1.3 were localized predominantly on chromatin. In prophase, HDAC3 and H1.3 partially co-localized with each other and partially co-localized with the chromosomes as well. However, during the remaining mitotic stages, HDAC3 and histone H1.3 were co-localized around but not on the condensed chromosomes. The co-localization of H1.3 and HDAC3 was very high, particularly in metaphase, anaphase, and telophase (Fig. 6, *B–D*). These results support the co-immunoprecipitation data and indicate that histone H1.3 is associated with HDAC3 *in vivo*. However, these two proteins were not localized on condensed chromatin during most stages of mitosis.

Although some reports have shown that an HDAC3 core complex is associated with microtubules and required for kinetochore-microtubule attachment (5), in our HDAC3 and histone H1.3 immunostaining of anaphase and telophase, we noticed that these proteins co-localized specifically between the separating sister chromatids at the spindle midzone and at the spindle poles (Fig. 6*C*, *Anaphase*). This implied the possibility that HDAC3-H1.3 complexes may be associated with polar microtubules that are not attached to chromosomes. To further investigate this possibility, we performed immunostaining with Eg5, a specific polar microtubule motor protein and a member of the mitotic kinesin family associated with assembly and maintenance of the mitotic spindle (23). Eg5 has been reported to be involved in cross-linking and anti-parallel sliding of polar microtubules (24, 25). To test our hypothesis that HDAC3 and H1.3 are co-localized on polar microtubules, immunostaining of Eg5 was performed along with either HDAC3 or H1.3 (Fig. 6). The confocal images showed a co-localization of H1.3 and HDAC3 with Eg5 during anaphase, which indicated that HDAC3 and H1.3 (and, therefore, the complex) are either localized on the polar microtubules or in proximity to them (Fig. 6*C*, *Anaphase*).

##### The HDAC3-H1.3 Complex Contains at Least Seven Proteins

**FIGURE 7.**
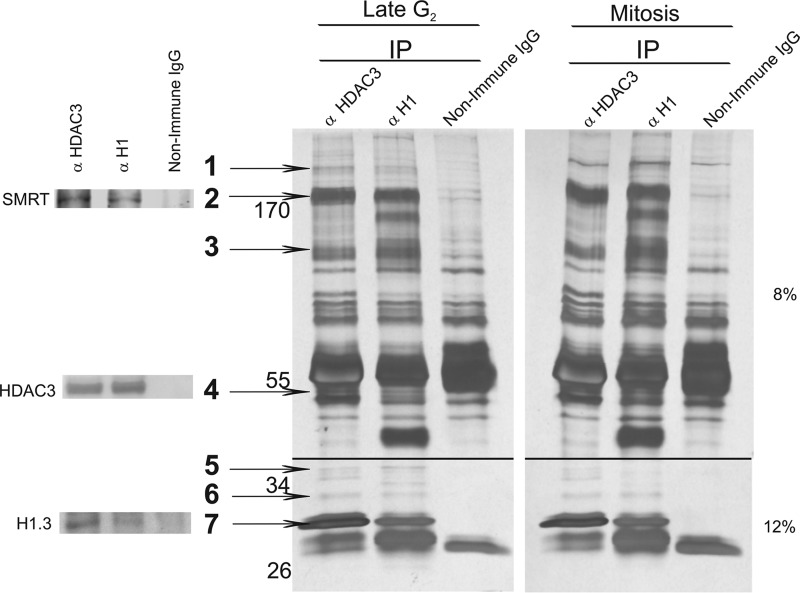
**The HDAC3-H1.3 complex contains at least five additional proteins.** Silver staining of proteins immunoprecipitated (*IP*) with anti-HDAC3, anti-histone H1, and non-immune IgG from HeLa S3 cell extracts from mitosis and late G_2_ phase. Proteins in the HDAC3-H1.3 complex were identified on the basis of the common bands (*numbered arrows*) that appeared in the histone H1 and HDAC3 immunoprecipitates but were not present in non-immune IgG immunoprecipitate.

To determine whether there are any additional proteins in the HDAC3-H1.3 complex and whether there are differences among the components of the complex between late G_2_ phase and mitosis, we immunoprecipitated the complex from late G_2_ phase and mitotic cell extracts using antibodies against HDAC3 and histone H1. The immunocomplexes were resolved on SDS-PAGE, and the protein bands were visualized by silver staining (Fig. 7). Common bands, precipitated by anti-HDAC3 and anti-histone H1 antibodies, which were absent from the non-immune IgG immunoprecipitate, were expected components of the HDAC3-H1.3 complex. Overall, seven such protein bands, numbered 1–7, were isolated from the immunoprecipitated complex. Bands representing HDAC3 (Fig. 7, *band 4*), H1.3 (Fig. 7, *band 7*), and SMRT (Fig. 7, *band 2*) proteins were verified by immunoblotting of a parallel silver-stained gel. The identities of the other four proteins in bands 1, 3, 5, and 6 are currently being determined. The silver staining and analysis of the complexes from mitosis and late G_2_ phase (Fig. 7) revealed identical patterns.

## Discussion

This report describes a novel protein complex containing HDAC3 and linker histone H1.3 that is activated during mitosis. Previously, SirT1 interaction with histone subtype H1.4 has been demonstrated to be involved in heterochromatin establishment in HEK293 cells (9). However, SirT1 belongs to the class III HDACs (sirtuins), which are structurally and functionally different from class I, II, and IV HDACs. Therefore, our study shows, for the first time, an association of a class I HDAC protein (HDAC3) with a linker histone, suggesting a wider role for HDAC3 and H1. Although several HDAC3 complexes have been isolated (particularly the N-CoR·SMRT complex (26, 27)), to the best of our knowledge, the mechanism of activation of HDAC3 in these complexes in relation to the cell cycle has not been studied. We hypothesized that the interaction between histone H1 and HDAC proteins correlates with mitotic events.

Co-immunoprecipitations and pulldown assays indicated a direct physical association between HDAC3 and H1.3 and demonstrated an increase of the level of this complex in late G_2_ phase and mitosis. HDAC3 did not interact with any other histone H1 variants. Further supporting the unique association of HDAC3 and histone H1.3, they co-localized partially during interphase into prophase and co-localized fully during the later stages of mitosis at the polar microtubules and spindle poles. Linker histone subtypes vary in their nuclear localization (28), ability to regulate gene expression (29), posttranslational modifications (10, 30), and affinity to chromatin (31). This wide range of differences between linker histone subtypes indicates their specific functions and can explain the exclusive interaction of HDAC3 specifically with histone subtype H1.3.

Previous studies have described the importance of HDAC3 in mitotic progression. RNAi-mediated knockdown of HDAC3 in HeLa cells causes cell cycle arrest in G_2_/M phase (4, 32), loss of H3S10 phosphorylation (a mitosis marker), and impaired mitotic progression such as incomplete chromosomal condensation and “lagging” chromosomes (4). In particular, HDAC3 knockdown in HeLa cells using siRNA showed smaller, collapsed mitotic spindles and excluded chromosomes from mitotic bipolar spindles. In addition, HeLa S3 cells showed premature sister chromatid separation in HDAC3 knockdown cells (2). Together, these results demonstrate the necessity of active HDAC3 in spindle formation and chromosomal alignment during mitosis (5). The importance of histone H1 for chromatin condensation during mitosis and in gene-specific transcription repression (9, 11, 33, 34) has also been well documented. Because of the role of histone H1 in chromatin folding, it is vital for mitotic condensation (35). Therefore, HDAC3 and histone H1 are both known to independently contribute to mitosis. Our analysis of the novel HDAC3-H1.3 complex supported a role for the complex in progression of mitosis. First, the HDAC3-H1.3 complex was present at higher levels during late G_2_ phase and mitosis. Second, the HDAC3-H1.3 complex isolated from mitotic cells was capable of deacetylating H3K9 *in vitro*. The late G_2_ phase complex lacked deacetylase activity. In synchronized cell extracts, both H3K9 and H4K5 residues showed low levels of acetylation during mitosis, consistent with hypoacetylation at these sites during mitosis, as reported previously (14). The mitotic HDAC3-H1.3 complex deacetylated only H3K9-Ac *in vitro*, suggesting that this complex may deacetylate H3K9-Ac, but not H4K5-Ac, during mitosis. H4K5-Ac may be deacetylated by a different HDAC or a different HDAC3 complex. Therefore, although the complex accumulated during late G_2_ phase, the complex was only activated during mitosis, and apparently its substrate specificity was changed from that of the HDAC3·N-CoR recombinant core complex.

We investigated the activation mechanism of the HDAC3-H1.3 complex isolated from late G_2_ phase by phosphorylating HDAC3 in the complex. Zhang *et al.* (22) have demonstrated the activation of HDAC3 upon its phosphorylation by CK2 *in vitro*. However, this work did not correlate HDAC3 activation by CK2 to a specific cell cycle stage, nor was it verified *in vivo*. Multiple studies have linked CK2 to mitotic progression, making it a logical candidate for activation of the HDAC3-H1.3 complex upon entry into mitosis. In *Saccharomyces cerevisiae,* CK2 is required during the G_2_/M phase transition (36). The regulatory subunit of tetrameric CK2 protein, CK2β, is phosphorylated by the mitotic kinase CDK1 in a cell cycle-dependent manner (37–39). CK2β has also been shown to regulate cell cycle progression at the beginning of mitosis (40). In our experiments, inactive HDAC3 in isolated complexes from late G_2_ phase cells could be activated *in vitro* by phosphorylating the HDAC3 with CK2. The mitotic, but not the late G_2_ phase, HDAC3-H1.3 complex showed significant phosphorylation of HDAC3 Ser-424. Furthermore, MCF-7 cell double knockdowns for CK2α and CK2α' showed a significant reduction in the level of HDAC3 Ser-424 phosphorylation, indicating that CK2 has a major role in activating HDAC3 *in vivo* in the HDAC3-H1.3 complex during mitosis. A recent work has established the role of HDAC3 in posttranslationally stabilizing G_2_/M phase cyclin-dependent kinase 1 (CDK1) (41). Therefore, we suggest a mitotic regulatory positive feedback loop where HDAC3 positively activates CDK1, which activates CK2(β), which, in turn, activates HDAC3 by phosphorylation.

Finally, immunocytochemistry studies demonstrated that, during interphase and prophase, HDAC3 and H1.3 were partially co-localized with chromatin, whereas, during metaphase to telophase, the complex was co-localized on the outer periphery of chromosomes. Our results are consistent with those of Bhaskara *et al.* (42), who have shown that HDAC3 is associated with chromatin during interphase and prophase but not during metaphase and anaphase in wild-type mouse embryonic fibroblasts, and those of Li *et al.* (4), who have shown that HDAC3 is localized to condensed chromosomes in prophase of HeLa cells. Because HDAC3 and H1.3 co-localized at the spindle poles and between separating chromosomes during anaphase, we hypothesized that the complex is localized on polar microtubules. A recent study from Ishii *et al.* (5) has indicated the presence of HDAC3 on microtubules in HeLa cells. In that study, HDAC3 was concentrated on the microtubules near the pole during prophase, whereas, during prometaphase, metaphase, and anaphase, HDAC3 was observed to spread over entire spindles and to be absent from the poles. During telophase, HDAC3 was diffused throughout the cytosol (5). However, this study did not specify the type of microtubules on which HDAC3 was localized. In our studies, we observed HDAC3 and histone H1.3 to be co-localized with the polar microtubule motor protein Eg5, especially at the center of anaphase and telophase cells, whereas sister chromatids were separating during anaphase. Therefore, our data supported that the HDAC3-H1.3 complex localized on or next to polar microtubules and that this complex was distinctive from other HDAC3 complexes already reported.

HDAC3 has been shown to have association with many proteins, including Aurora B kinase (4), SMRT (20), N-CoR (20, 43), TBL1 (4, 20, 44), TBLR1 (44), CREB-binding protein (45), GATA-2 (46), MEF2 (47), and transcription factor TFII-I family proteins (48). To determine whether the mitotic and late G_2_ phase complex components differ, we analyzed the silver staining pattern of the HDAC3·H1.3·SMRT·N-CoR complex, which revealed at least four potentially additional proteins. SMRT and N-CoR have been reported previously to function as co-repressors for HDAC3 (19) and are well known to mediate repression for various transcription factors (49). HDAC3 alone does not have deacetylase activity. However, the deacetylase-activating domain of SMRT has been shown to physically interact with HDAC3 and to activate the deacetylase function of inactive HDAC3 enzyme (19). The presence of SMRT and N-CoR in the presumptive microtubule-localized HDAC3-H1.3 complex could be an indication of the probable role of HDAC3 as a deacetylase not only in the chromatin environment but also in the context of microtubules. Several HDAC inhibitors have been shown to cause impaired chromosomal separation (4, 50–52). This could be a result of weakened microtubule function, which may be an effect of HDAC inhibition, or, as shown recently, because of hyperacetylation of α-tubulin by inhibition of HDAC6 and SirT2 (class III HDAC) (53). Nakayama *et al.* (54) have showed that histone H1 is associated with and even organizes microtubules in tobacco BY-2 cells. However, no studies have evaluated the possibility of histone H1 having a role in cytoskeleton maintenance. Overall, the presence of histone H1 in the complex could play a role in the localization and possible binding of the complex to polar microtubules or microtubule-associated proteins. We considered the possibility of HDAC3 deacetylating tubulin on the basis of the localization of the complex to polar microtubules. However, Ishii *et al.* (5) have already reported that HDAC3 is unable to deacetylate α-tubulin. The possibility of HDAC3 deacetylating β- or γ-tubulin or one of the microtubule-associated proteins still remains to be investigated. Previously, several multiprotein complexes that include HDAC3 and N-CoR and that play a role during mitosis have been identified and characterized (4, 5). Our complex is unique because it contains H1.3, unlike any other HDAC3 complexes reported previously. In addition, the HDAC3-H1.3 complex components are distinct from the mitotic HDAC3 complex reported by Li *et al.* (4) that contained A-kinase anchor protein 95 (AKAP95) and homologous to AKAP95 (HA95), suggesting the identification of a novel complex that may have a distinct function. The different mitotic HDAC3-AKAP95-HA95 complex identified by Li *et al.* (4) may be explained by the different cell line (HeLa S3 *versus* 293T), the lack of cell synchronization, or the use of ectopically expressed FLAG-HDAC3.

Further functional and structural analysis of the HDAC3-H1.3 complex is needed to link HDAC3 to a potential deacetylation of tubulin or microtubule-associated proteins via its recruitment by H1.3 or other complex factors. Deacetylation of core histones may be an important step in the condensation of chromatin at the beginning of mitosis and proper mitotic progression. We suggest that the HDAC3-H1.3 complex might be involved in a variety of mitotic functions, such as H3 deacetylation and chromatin compaction, regulation of microtubules dynamics, and tethering histone H1.3 out of the condensed chromosomes. A detailed study of the functionality of such an association may help us to understand the mitotic process and provide new targets, such as HDAC3, H1.3, and other complex components, for cancer therapy. It is interesting to note that H1.3 has been reported recently to be implicated in ovarian cancer (55, 56) and pluripotent ES cell growth (57).

## Author Contributions

M. B., H. P., and C. W. conceived and designed the study. M. B., H. P., C. W., H. C. W., and S. D. wrote the manuscript. H. P., C. W., R. W. G., and S. D. designed and performed the experiments, analyzed the data, and prepared the figures for publication. M. B. and H. C. W. helped to analyze the data and contributed ideas for the research. All authors reviewed the results and approved the final version of the manuscript.

## Footnotes

2 The abbreviations used are:

HDAC: histone deacetylase
CREB: cAMP response element-binding protein
SMRT: silencing mediator of retinoic acid and thyroid hormone receptor
N-CoR: nuclear receptor corepressor 1
H3K9-Ac: acetylated histone H3 lysine 9.
